# Assessing the Utility of the DAPT Score and PRECISE-DAPT Score in Determining the Appropriateness of Dual Antiplatelet Therapy in Patients With Acute Myocardial Infarction/Percutaneous Coronary Intervention

**DOI:** 10.1155/2024/1489008

**Published:** 2024-10-30

**Authors:** Abhishek Singh, M. A. Hussain, Shyam Chand Chaudhary, Akriti Bharadwaj, K. K. Sawalani, Akshyaya Pradhan, Rishi Sethi

**Affiliations:** ^1^Department of Cardiology, King George's Medical University, Lucknow 226003, India; ^2^Department of Internal Medicine, King George's Medical University, Lucknow 226003, India

**Keywords:** dual-antiplatelet therapy, hemorrhage, myocardial infarction, ST elevation myocardial infarction

## Abstract

**Background:** Utilizing the two available prediction models, i.e., the dual antiplatelet therapy (DAPT) score and predicting bleeding complication in patients undergoing stent implantation and subsequent dual antiplatelet therapy (PRECISE-DAPT) score, we aimed to determine the appropriateness of the DAPT in patients with acute myocardial infarction (AMI) in patients undergoing percutaneous coronary intervention (PCI).

**Methods:** We retrospectively enrolled 235 patients of AMI and for all the patients and thorough information regarding history, risk factors, and medications were collected. Both DAPT and PRECISE-DAPT scores were calculated. The patients were divided by their recommended cutoffs and the appropriateness of the duration of the recommended DAPT was measured based on the observed scores. Bleeding academic research consortium (BARC) classification was used to define the bleeding events. In the patients with DAPT score ≥ 2 and PRESICE-DAPT < 25, the prolonged use of DAPT was recommended.

**Results:** Overall, 235 patients, predominantly male (78.7%), with baseline characteristics exhibiting high rate of smoking (31.1%), diabetes (35.3%), and hypertension (32.8%) were found. The widely prescribed DAPT combination was aspirin with clopidogrel (72.3% at discharge and 46% on current use). Among all the enrolled patients, 163 patients were on DAPT while 71 were on single antiplatelet therapy (SAPT). A significant association between DAPT and PRECISE-DAPT scores was noted in terms of SAPT and DAPT. The appropriateness of DAPT was checked based on the scores, where 81% of the patients with DAPT ≥ 2 and 77.24% with PRECISE-DAPT score < 25 were appropriately prescribed with DAPT. The primary reason for drug interruptions was self-advised. The incidence of bleeding events was observed to be 7.23%, among which 5.1% had Type 1 bleeding according to BARC.

**Conclusion:** Both DAPT and PRECISE-DAPT scores could be used to determine the appropriateness of the recommendations of DAPT in patients with AMI or undergoing PCI.

## 1. Introduction

Despite current improvements in percutaneous coronary intervention (PCI) techniques and devices, the patients with acute myocardial infarction (AMI) continue to have a higher mortality risk compared with the general population [1, 2].

Dual antiplatelet therapy (DAPT), which is a combination of aspirin with a P2Y12 inhibitor, is extensively used in treating AMI. The DAPT has shown superior efficacy in preventing the recurrence of major adverse cardiovascular events (MACEs) among individuals who have been diagnosed with AMI and are undergoing PCI, compared with those on aspirin only [3, 4]. The recommendations from the American College of Cardiology (ACC)/American Heart Association (AHA) and the European Society of Cardiology (ESC) guidelines suggest the use of DAPT as a beneficial preventive approach for patients discharged following acute coronary syndrome (ACS), irrespective of the specific type of revascularization procedure performed [5]. However, the utilization of DAPT is complicated by its associated risk of bleeding, leading to ongoing debates about the optimal duration of therapy [6]. Also, among patients with thrombocytopenia, there are certain concerns over DAPT about increased bleeding risk, and hence single antiplatelet therapy (SAPT) is usually preferred in such cases [4].

Before starting DAPT therapy, a patient's risk profile must be thoroughly assessed to determine the appropriate duration of treatment [6]. Literature indicates that older age, higher Killip class, increased heart rate, elevated blood pressure, raised levels of serum creatinine, increased white blood cell counts, and low hemoglobin levels have been strong predictors of in-hospital and early death in patients diagnosed with AMI undergoing PCI [7, 8]. Based on these considerations, the DAPT and PRECISE-DAPT risk prediction models have been developed to help determine the appropriate duration of DAPT. First recommended in the 2017 ESC guidelines, these scores can aid in identifying patients at a higher risk of thromboembolism and a lower risk of bleeding [9]. In addition, their accuracy and validity have been tested in various patient populations [6, 10]. This study aims to evaluate the appropriateness of DAPT for individuals with AMI using the DAPT and PRECISE-DAPT scoring systems.

## 2. Materials and Methods

The current study was designed retrospectively, and we examined the medical information through patient medical records. A total of 235 patients who were diagnosed with AMI with or without STEMI and/undergoing PCI were enrolled in the study. Patients were excluded if they had chronic illness, were pregnant, or had bleeding diathesis. The study was performed after obtaining approval from the ethics committee (Ref no: 102^nd^ ECM II B-Thesis/P115). The study was conducted in accordance with the Declaration of Helsinki.

Comorbidities and past medical information were obtained from all patients, including age, gender, cardiovascular risk factors, procedural outcome, and antiplatelets regimen. For all the patients, the two risk prediction scores, i.e., DAPT and PRECISE-DAPT scores were calculated. Bleeding events were defined as per bleeding academic research consortium (BARC) classification.

The DAPT score ranges from −2 to 10 points. The calculation of the DAPT score considers nine variables. In detail, −2 points were given for age ≥ 75 years, while 1 point for age between 65 and 75 years, 0 point for age < 65 years, 1 point each for cigarette smoking, diabetes mellitus, myocardial infarction (MI) at presentation, prior PCI or prior MI, paclitaxel-eluting stent and stent diameter < 3 mm, and 2 points for vein graft stent and congestive heart failure (CHF) or left ventricular ejection fraction (LVEF) < 30%, respectively. The total score was determined by summation of all points of the 9 predictive risk factors. While the PRECISE-DAPT scoring system (ranges from 0 to 100 points) comprises five variables, i.e., age, creatinine clearance, hemoglobin, white blood cell count, and previous spontaneous bleeding. The PRECISE-DAPT score < 25 shows that the patient had low bleeding risk. Prolonged duration DAPT is recommended for patients with a DAPT score ≥ 2 due to higher ischemic risks, and in contrast, short duration DAPT (3–6 months) is recommended for patients with a PRECISE-DAPT score ≥ 25 because of higher bleeding risks.

All the statistical analysis was performed using SPSS Version 21.0. The categorical variables were expressed as the mean ± standard deviation and the quantitative data were presented as the frequency or percentage. The correlation between the variables was analyzed using a chi-square test. The *p* value < 0.05 was considered to be statistically significant.

## 3. Results

Among 235 patients, the majority (78.7%) were male and the highest proportion (36.2%) was within the 60–69 years of age group. The baseline characteristics of these patients are detailed in Table 1. Notably, 31.1% were smokers, 35.3% had diabetes mellitus, and 32.8% had hypertension. Specific findings included 29 patients with LVEF < 30%, 45.1% diagnosed with multivessel disease, and 27.7% underwent multiple stent implantations. The average levels of hemoglobin, serum creatinine, and estimated glomerular filtration rate (eGFR) in the study group were 12.7 ± 1.88 g/dL, 1.11 ± 0.50 mg/dL, and 71.19 ± 24.58 mL/min/1.73 m^2^, respectively.

**Figure 1:**
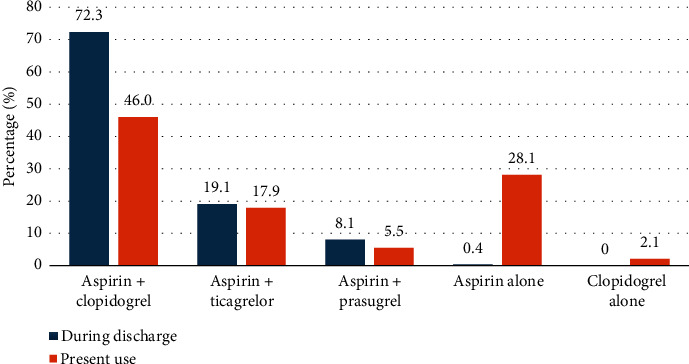
Distribution of antiplatelet drugs used in the study groups.

**Figure 2:**
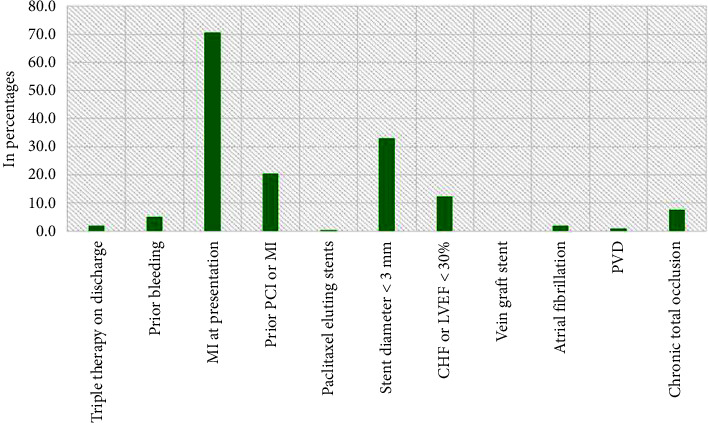
The distribution of cases according to variables used in risk scores. CHF, congestive heart failure; LVEF, left ventricular ejection fraction; MI, myocardial infarction; PCI, percutaneous coronary intervention; PVD, peripheral vascular disease.

**Figure 3:**
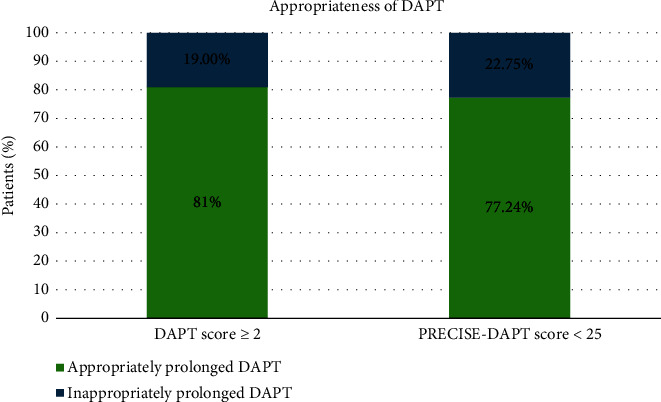
Appropriateness of antiplatelet therapy according to DAPT and PRECISE DAPT scores.

The distribution of the antiplatelet drugs used in the study population is illustrated in Figure 1. Aspirin with clopidogrel was the widely prescribed combination of DAPT, followed by aspirin with ticagrelor and aspirin with prasugrel. However, 28.1% and 2.1% were on single antiplatelet drug aspirin and clopidogrel, respectively. The DAPT and PRECISE-DAPT scores determined based on the variables such as MI at presentation, prior bleeding, prior PCI, and paclitaxel-eluting stents, as described in Figure 2, revealed that majority of the study population demonstrated presented with MI at the time of admission (70%).The patients were categorized into two groups based on the number of antiplatelets regimen, and 71 patients were on single antiplatelets agents while 163 out of 235 were on DAPT. In addition, the relationship between DAPT and PRECISE-DAPT scores within these groups was examined. The analysis in Table 2 exhibited that patients receiving DAPT had a higher prevalence of DAPT score ≥ 2 and PRECISE-DAPT score < 25. Both DAPT score and PRECISE-DAPT scores were found to be significantly associated with SAPT and DAPT therapy. In total, 81% patients with DAPT score ≥ 2 and 77.24% patients with PRECISE-DAPT score < 25 were found; the DAPT was appropriately recommended for prolonged period of time (Figure 3).

**Figure 4:**
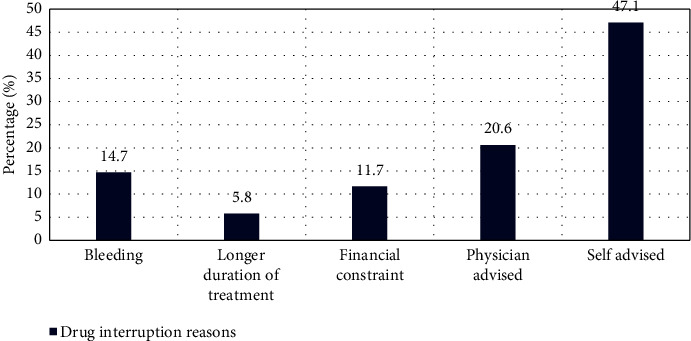
Representation of drug interruption reasons.

Out of 17 patients who had bleeding, 12 experienced minor bleeding, classified as BARC bleeding Type 1. None of the patients taking SAPT had any bleeding incidents. No significant association of BARC bleeding type was found with the type of antiplatelet drug used (*p*=0.976). Approximately, 15.8% of the cases involved drug interruption, with self-advised reason being the primary cause of it (47.1%), as illustrated in Figure 4.

## 4. Discussion

The main targets for the secondary prevention of an AMI are platelet activation, adhesion, and aggregation. The use of evidence-based DAPT therapy is of utmost importance, especially in managing patients with AMI. In present study, we found that the DAPT score and PRECISE-DAPT score could be used in assessing the utility of DAPT.

In the present study, we observed male predominance (78.7%) among all the patients. Similar gender dominance was observed in a study by Lu et al. [4]. A higher prevalence of various risk factors such as diabetes mellitus, hypertension, and smoking were observed in our study. Similar results were observed in a study performed by Chen et al., which compared data based on dual antiplatelet and triple antiplatelet therapies in patients with AMI and are undergoing PCI, where patients are on DAPT therapy, 45.3% had hypertension, 49.2% had habit of smoking, and 24.5% had diabetes mellitus [11].

The conflict between antiplatelet therapy and bleeding risk has been a concern since long. In 2017, the ESC guideline provide the official recommendation for DAPT, highlighting the significance of two scoring systems, i.e., the DAPT score and PRECISE-DAPT score, to aid decision making of DAPT [9]. The DAPT score incorporates both clinical and procedural variables and can guide the extension of DAPT for upto 30 months if the score is two or higher [12]. Parallelly, the PRECISE-DAPT score, which combines clinical and laboratory factors, recommends the shortening of DAPT duration if the score is greater than or equal to 25 [13].

In a study conducted in Denmark involving 28,449 patients who survived a first AMI between 2009 and 2012, a slight increase was noted in the percentage of patients receiving DAPT from 68% in 2009 to 73% in 2012 [14]. This trend is consistent with data from several cardiovascular disease registries, which report overall usage of DAPT among patients discharged after an ACS in Europe and Canada between 60% and 80% [15, 16]. Based on these findings, it is recommended that at least 75%–85% of the patients with AMI should be treated with DAPT, unless they are at high risk of bleeding. In our study, we observed that 70.6% of the patients were on DAPT and had DAPT score ≥ 2, which was statistically significant (*p* < 0.001). These findings correlate well with the abovementioned data. In our study, 69.3% were adhering to DAPT after 1 year of AMI/PCI. A study by Mehta SR and colleagues suggests that patients who are not at high risk of bleeding should continue DAPT beyond 1 year, which is consistent with Canadian Cardiovascular Society guidelines [17]. DAPT-related bleeding complication is the most commonly observed after coronary stent implantation and are linked to higher mortality rates, compromised quality of life, and increased healthcare expenses [18, 19]. The PRECISE-DAPT score is a standardized tool used for predicting out of hospital bleeding during DAPT therapy [13]. In addition, Gragnano and colleagues found that the PRECISE-DAPT score can serve as a bleeding risk prediction model for patients on SAPT [20]. According to a study by Choi et al., the PRECISE-DAPT score may aid in determining the individual duration of DAPT after PCI in patients diagnosed with ACS [21]. In our study, a large number of patients on SAPT had PRECISE-DAPT score ≥ 25 whereas 68.7% of those on DAPT had PRECISE-DAPT score < 25, and this finding is consistent with the study done by Lu et al. [4].

Antiplatelet therapy is crucial for preventing complication in patients with AMI undergoing PCI. The combination of aspirin and clopidogrel was most commonly used. In the study by Ke et al., it was concluded that high doses of aspirin/clopidogrel in patients with high DAPT scores are associated with reduced mortality of AMI [22]. A study included 225 patients having a higher DAPT score (≥ 2) and these patients were stratified into two groups, i.e., only aspirin vs. aspirin and clopidogrel. This study found that patients with a high DAPT score did benefit from prolonged use of DAPT (> 1 year) through a reduction in MI but reported more bleeding events [22].

In the present study, we also assessed the drug adherence of the patients. The most common reason for interruption was self-advised observed in 47.1% of the patients, where patients have stopped their medication by their own choice without any reason given to the physician. In the current study, self-advised drug interruptions were more common, likely due to socioeconomic factors and patients' perception of not needing medication if they felt better without consulting a physician. Addressing these issues through increased education and communication could help mitigate this problem. These findings were supported by a study done by Melloni et al. [23]. However, contrasting results were observed in a study conducted by Mehran et al. [24]. In future, large and adequately powered prospective studies are better poised to throw light on the utility these scores.

### 4.1. Limitations

There are some limitations of the study that need to be listed. First, the study was conducted at a single center and was observational and retrospective in nature, hence generalizability of the findings is limited. In addition, our study did not report various types or dosages of P2Y12 inhibitors used. We lacked a thorough analysis of the adverse effects from any culprit lesions that were not treated. We did not perform multivariable analysis among different drug interruption reasons. Also, the length of DAPT and subgroup analysis for the same is very interesting issue but was not performed in the present study. Further larger studies are warranted to evaluate the utilization of both the scores in patients having AMI.

## 5. Conclusion

In conclusion, a significant association was observed between PRECISE-DAPT and DAPT scores with SAPT and DAPT therapies. These scores may help to identify patients for whom the prolonged DAPT would be beneficial outweighing the risk. Therefore, these scoring systems would aid in making clinical decision regarding the appropriate duration of DAPT in patients with AMI.

## Figures and Tables

**Table 1:** Baseline characteristics of the study population.

Variable		Patients (*n* = 235)
Age	30–39 years, *n* (%)	8 (3.4%)
	40–49 years, *n* (%)	39 (16.6%)
	50–59 years, *n* (%)	71 (30.2%)
	60–69 years, *n* (%)	85 (36.2%)
	≥ 70 years, *n* (%)	32 (13.6%)

Sex	Male, *n* (%)	185 (78.7%)
	Female, *n* (%)	50 (21.3%)

Risk factors	Diabetes mellitus, *n* (%)	83 (35.3%)
	Hypertension, *n* (%)	77 (32.8%)
	Smoking, *n* (%)	73 (31.1%)
	Tobacco, *n* (%)	77 (32.8%)
	Others (family history, dyslipidemia, and obesity), *n* (%)	6 (2.6%)

CHF or LVEF < 30%, *n* (%)		29 (12.3%)
Multivessel disease, *n* (%)		106 (45.1%)
Multiple stents implanted, *n* (%)		65 (27.7%)
Complex bifurcation lesion, *n* (%)		9 (3.8%)
Total stent length > 60 mm, *n* (%)		34 (14.5%)
Chronic total occlusion intervention, *n* (%)		18 (7.7%)
Hb (g/dL, mean ± SD)		12.70 ± 1.88
Serum creatinine (mg/dL, mean ± SD)		1.11 ± 0.50
eGFR (ml/min/1.73 m^2^, mean ± SD)		71.19 ± 24.58

*Note:* Data are represented as *n* (%) and mean ± SD as appropriate. *p* < 0.05 was considered statistically significant.

Abbreviations: CHF, congestive heart failure; eGFR, estimated glomerular filtration rate; Hb, hemoglobin; LVEF, left ventricular ejection fraction.

**Table 2:** Association of DAPT and PRECISE-DAPT scores with single/double antiplatelet drug use.

Variable		Drugs		Chi square	*p* value
		SAPT	DAPT		
		Patients (*N* = 71)	Patients (*N* = 163)		
DAPT score	≥ 2	27 (38.00%)	115 (70.6%)	21.93	**< 0.001**
	< 2	44 (62.00%)	48 (29.4%)		

PRECISE-DAPT score	≥ 25	38 (53.50%)	51 (31.30%)	10.37	**0.001**
	< 25	33 (46.50%)	112 (68.70%)		

*Note:* Data are represented as *n* (%) as appropriate. *p* < 0.05 was considered statistically significant. Bold values mean statistically significant (*p* < 0.05).

Abbreviations: DAPT, dual antiplatelet therapy; PRECISE-DAPT, predicting bleeding complications in patients undergoing stent implantation and subsequent dual antiplatelet therapy; SAPT, single antiplatelet therapy.

## Data Availability

The data used to support the findings of this study are available from the corresponding author upon reasonable request.
